# The influence of upward social comparison on retail trading behaviour

**DOI:** 10.1038/s41598-023-49648-3

**Published:** 2023-12-19

**Authors:** Sandra Andraszewicz, Dániel Kaszás, Stefan Zeisberger, Christoph Hölscher

**Affiliations:** 1 Chair of Cognitive Science, ETH Zurich, Clausiusstrasse 59, 8092 Zürich, Switzerland; 2 Future Resilient Systems, Singapore-ETH Centre, 1 CREATE Way, #06-01 CREATE Tower, Singapore, 138602 Singapore; 3 Institute for Management Research, Radboud University Nijmegen, Heyendaalseweg 141, 6525 Nijmegen, The Netherlands; 4 Department of Banking and Finance, University of Zurich, Plattenstrasse 32, 8032 Zürich, Switzerland

**Keywords:** Human behaviour, Psychology and behaviour

## Abstract

Online investing is often facilitated by digital platforms, where the information of peer top performers can be widely accessible and distributed. However, the influence of such information on retail investors’ psychology, their trading behaviour and potential risks they may be prone to is poorly understood. We investigate the impact of upward social comparison on risk-taking, trading activity and investor satisfaction using a tailored experiment with 807 experienced retail investors trading on a dynamically evolving simulated stock market, designed to systematically measure various facets of trading activity. We find that investors presented with an upward social comparison take more risk and trade more actively, and they report significantly lower satisfaction with their own performance. Our findings demonstrate the pitfalls of modern investment platforms with peer information and social trading. The broad implications of this study also provide guidelines for improving retail investor satisfaction and protection.

## Introduction

More and more people worldwide are using online financial services^1^ making digitization significantly shape the environment in which people invest their money. While ‘investing in speculative assets is a social activity’^2^, online tools have made the role of social influence in financial investment even more prominent^3^. Social trading is a new form of investing combining social media and investing (see the Online Appendix for examples) that often makes the information of peer top performers widely accessible. In this means, less experienced investors may benefit from learning from more experienced investors^4,5^. Information distributed via social trading platforms can result in increased performance of portfolios focusing on clear geographical areas^6^. Also, being observed on social trading platforms diminishes the disposition effect: a common bias in financial investing in which investors sell the best performing assets and hold on to the loss-generating asset^7,8^. On the other hand, social trading exposes investors to upward social comparison: the process of comparing oneself to better peers. Research indicates that social influence results in irrational and impulsive trading behaviour^9–17^. In retail trading, inability to resist impulses is linked to higher trading activity, worse financial outcomes^18^, lower portfolio diversification related to worse risk management, and higher likelihood to seek financial advice^19^.

Excessive risk-taking has often been investigated as a typical symptom of irrational trading^11,14–16,20^. However, observed risk-taking behaviour can be driven by diverse cognitive processes, leading to varying risk preferences^21^. Relatively few studies have investigated trading activity such as trading frequency^14,22^, portfolio turnover^18,19^ and volume^22^. Different facets of trading activity may indicate different psychological drivers of behaviour. For example, if an investor repeatedly conducts small-volume transactions, such as buying and selling one stock, their behaviour is likely to be unplanned and driven by their emotional state or affect. In contrast, generally executing large-volume trades is likely to be motivated by a trading strategy resulting from a cognitive process and executing many high-volume transactions could indicate that an investor’s emotional state keeps them alert to execute a planned trading strategy^23^. Affective reactions are fast, emotional and impulsive, whereas cognitive reactions are slower, planned, and reasoned^24^.

Consequently, the psychological mechanisms underlying trading behaviour in social environments require the investigation of trading activity^25–27, p. 259]^ and this is now possible through the large quantities of data available on online trading platforms^28^. In this study, we bring together three aspects. First, we investigate the impact of upward social comparison on several aspects of trading behaviour by systematically measuring risk-taking and trading activity in an environment that mimics a recent financial technology trend. Second, we demonstrate that experienced retail investors exposed to outcomes of top traders have lower satisfaction from their performance and increase the fraction of risky assets in their portfolios. Third, we show that they increase different facets of trading activity in heterogeneous ways, suggesting that online trading activity could help explain the drivers of the trading behaviour resulting from social influence.

‘Social influence is defined as change in an individual’s thoughts, feelings, attitudes, or behaviours’^29^. Social comparison is a form of social influence in which an individual changes their behaviour, judgment or attitudes as a result of observing others’ behaviour. Social comparison refers to people’s tendency to compare themselves with others^30^, to reduce uncertainty about themselves by evaluating their abilities^31^. In goal-directed behaviour, people compare their own performance to a reference group that is better than they are, in upward social comparison, to increase their performance, or to a worse reference group, termed downward social comparison, to preserve or boost their self-perception^32^. Both mechanisms constitute systems that regulate motivation to act and degree of present and future effort. They can also result in an emotional response. Feeling worse can trigger negative affect, whereas feeling better than others can trigger positive affect^33^.

Information about others’ performance sets a social reference point (SRP), and this can influence what individuals aspire to have even if this aspiration deviates from what they can rationally hope to obtain^16^. This aspiration, motivated by the desire to get ahead of others, influences risk-taking in investment tasks independently of whether the goal is achievable or not^15,16^. SRP influences investment risk-taking in tournament experiments, which list investors in a performance-based rank (see^34^ for a systematic review). A tournament is characterized by two motivator components: a monetary reward that depends on rank position and a nonmonetary incentive to outperform peers. Nonmonetary tournament components increase risk-taking only in underperforming professionals who report valuing socially relative performance more than monetary performance, whereas underperformers generally take more risk than overperformers^35^.

Presenting social comparison information leads to higher risk-taking, even when it is irrelevant to the payoff and not attached to tournament incentives^12^. Kirchler et al.^36^ experimentally showed that inducing upward social comparison through presenting financial outcomes of top investors increased risk-taking in an asset allocation task. Individuals also adjust their risk-taking levels to that of a better player in two-player investment games where one outperforms the other^37^. Such adjustments also occur beyond tournaments: investors adjust the risk of their portfolios when they receive bonuses dependent on a market index that indicates the performance of all market players^13^. A possible explanation of these findings is that relative performance implicitly creates a tournament reward structure^38^.

An experimental asset market is an experimental simulation of a financial market in which several humans trade artificial assets with each other using computer software. In such experiments, upward performance comparison^38^ and larger wealth inequality among market players^39^ leads to higher market prices and more inflated market bubbles. Also, novice investors underperform when their peers report very high returns^20^. A potential driver of this observation could be that sharing of the most successful performances encourages risky stock trading behaviour, especially in less experienced investors. Likewise, selectively communicating only positive information to investors causes higher stock market participation rates and consequently higher risk-taking^40^.

Copy trading is a form of social trading in which top social traders, who usually exhibit high trading performance, take the role of signal providers and are followed by other users. Research indicates that followers of the eToro trading platform are prone to take more risk, and they tend to overreact when the signal providers take more risk^5^, while followers on a European social trading platform increase their activity after signal followers had posted comments^10^. Experiments about copy trading found that information about the success of other investors leads to increased investment risk-taking, where participants choosing the riskiest asset are much more likely to copy this decision from another player than when choosing any of the less risky assets^41^. Investors also report that social influence through peer recommendations generally impacts their trading frequency and volume^22^. On social trading platforms, social influence can override rational decisions, raising questions about the use of these platforms^5^. This is especially important because followers prefer more-risky high-volatility stocks and trade more frequently, but obtain more negative returns^14^. A possible explanation for this observation is that that the followers perform less well because trades made after the signal is provided result in a lower performance than before-signal trades^14^.

Upward social comparison not only impacts how people behave but also how they feel. Comparing oneself to richer people decreases people’s life satisfaction^42^. Upward social comparison in financial investment results in lower satisfaction with one’s performance^38^, while engaging in online upward social comparison decreases positive affect^43^. Thus, affective and cognitive drivers of behaviour interplay^44–46^, such that affect moderates cognitive processes preceding actions^47^. Consequently, peer effect in risk-taking behaviour, such as investing, is a combination of conscious informed choices influenced by peers motivating individuals to execute particular strategies and affect arising from social comparison^48^.

However, data beyond risk-taking is needed to capture the psychological drivers of financial decisions because the timing of these decisions plays a crucial role^49^. Various aspects of trading activity, including trading frequency, average transaction volume and cumulative volume of all trades, can provide a much rounder picture of the potential psychological drivers of trading behaviour^25^. In dynamic decision tasks such as trading and investment, reaching the goal of generating high positive returns requires a series of decisions which need to be taken at the right times. The consecutive decisions are dependent on each other, such that earlier decisions determine the context and options available in subsequent decisions^50^. This causes the state of each decision to change continuously^51,52^. Experimental designs that incorporate these features and enable measurement of trading activity in a realistic and controlled way are scarce^53^.

Overall, the literature reviewed here indicates that social comparison can result in increased risk-taking in trading and investment^5,11–16,25,36,40,41^ and increased trading activity^5,9,10,18,25^ measured in various ways which are rarely linked to interpretations of cognitive and affective interpretations of these goal directed behaviours. Some studies^19,22^ investigated trading activity, but not in the context of social influence. Also, previous literature indicates that upward social comparison may be linked to a lower satisfaction from one’s own financial and economic outcomes^38,42^. To gain a better understanding of the effects of upward social comparison on trading behaviour,we programmed a dynamic artificial stock market substantially extending the features of^17^ to test two hypotheses preregistered on the Open Science Framework (https://osf.io/we48d/). Our hypotheses derived from the literature discussed here relate to risk-taking and three facets of trading activity:

### ***Hypothesis 1***

Upward social comparison will lead to increased risk-taking, where risk-taking is defined as the mean fraction of the risky asset in a portfolio throughout one experimental round.

### ***Hypothesis 2***

Upward social comparison will lead to increased trading activity:

- Leading to a larger number of transactions, where the number of transactions refers to the number of separate executed trades in one experimental round,
- Leading to higher volume of each individual trade, where the volume corresponds to the mean quick-trade button size, and
- Leading to higher overall trading volume, where the overall trading volume refers to the cumulative volume of all shares traded in one round.

Hypothesis 2c is not a direct mathematical composite of hypotheses 2a and 2b, but it embeds them.

## Results

**Figure 1:**
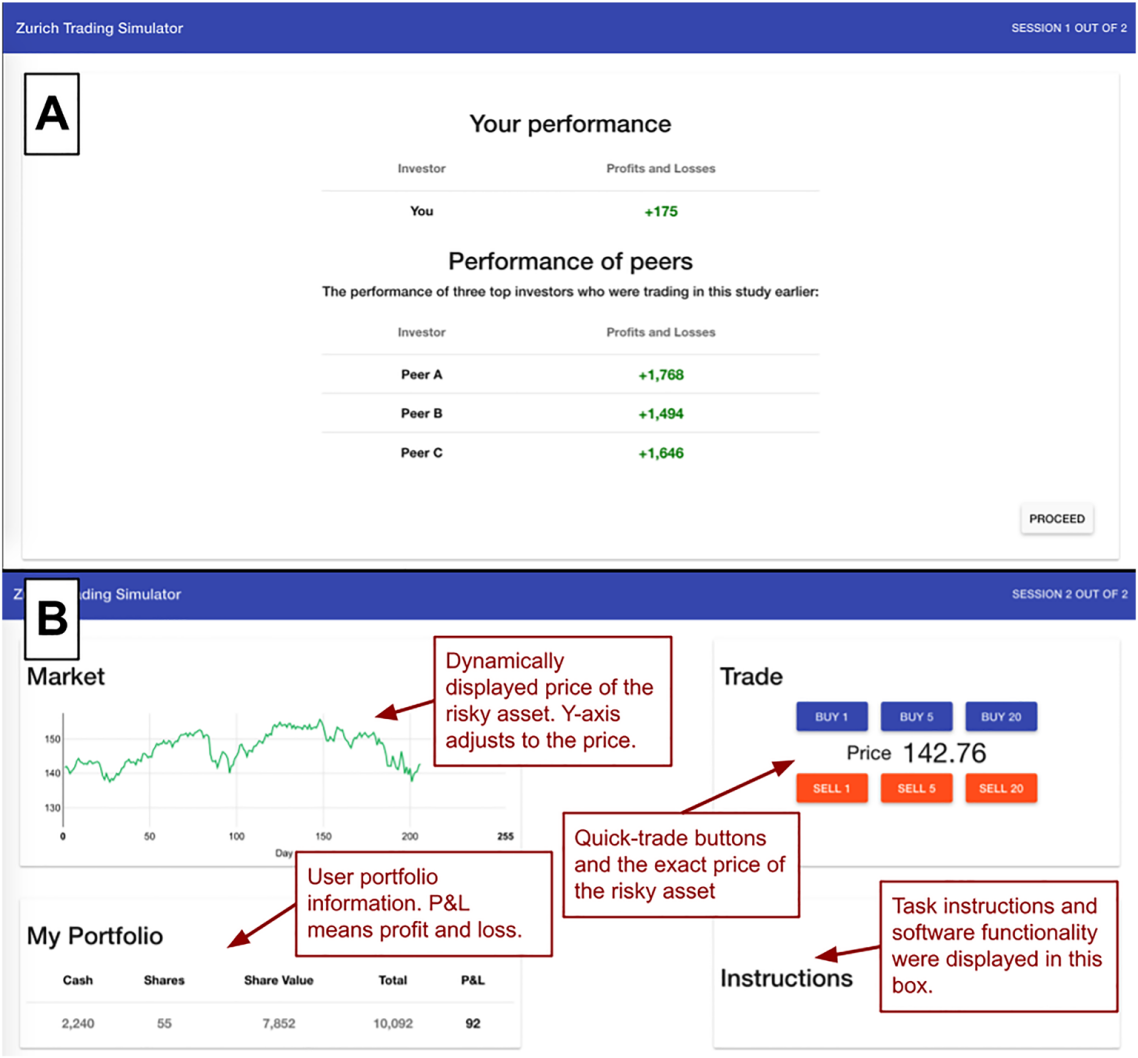
Social comparison information (**A**) and user interface of the trading task (**B**).

We developed a task that presented participants with historical day closing stock prices every 0.8 s (see Fig. 1) in two trading rounds. At the start of each round, their endowed capital was split equally between stocks and cash. In each round, our online experimental task displayed tick-by-tick 252 consecutive prices of one stock and enabled a participant to continuously buy and sell their shares in various volumes without transaction fees using six quick-trade buttons. On online platforms, buy buttons are a call-to-action feature^54,55^ that moderates the execution of a transaction. In our task, the buttons were designed to measure trading activity in three ways: (1) number of transactions, (2) average trading volume, and (3) overall volume of all traded shares. Participants could sell as many shares as they owned, and they could purchase as many shares as they wanted if they had enough cash. After the first round, participants in the experimental condition received information about the performance of three other participants in addition to feedback about their own performance (see Fig. 1). The three other participants were first batch study participants who obtained very high earnings. Participants in the control condition only received feedback about their own performance but not about their peers. Following the trading task, we measured participants’ satisfaction about their performance in the task. This experimental design extends the asset allocation design by^36^ enabling measuring the influence of upward social comparison on trading activity.

In this difference-in-difference design with prescreened, financially literate participants, we first tried to replicate previous findings that upward social comparison increases risk-taking (c.f., *Risk*). Then, we aimed to substantially extend previous findings by investigating the impact of upward social comparison on trading activity and trading satisfaction in experienced retail investors. We systematically measured trading activity that may indicate affective drivers of trading behaviour by the number of transactions (c.f., *Trans*); we also measured cognitive drivers by average trading volume (c.f., *Vol*), and the mediation of cognitive drivers by affect by the overall volume of all traded shares (c.f., *Vol_Sum*).

### Hypothesis testing

**Figure 2:**
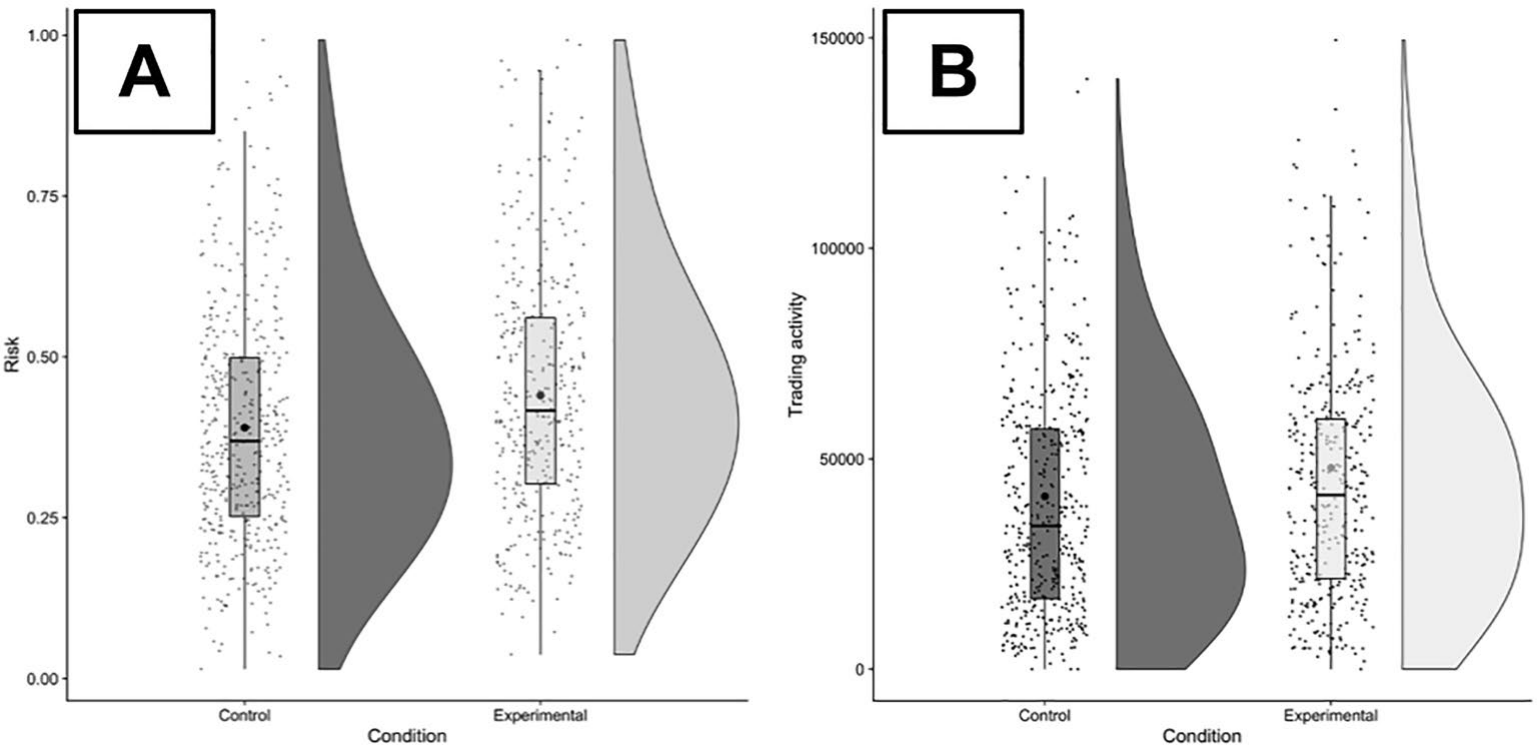
The distribution of portfolio risk between conditions (**A**) and trading activity expressed as *Vol_Sum* (**B**) in round 2*.* *Note* Horizontal bars indicate the group median, while larger dots indicate group means. In panel B, due to the presence of outliers the y-axis is bounded at 150,000 experimental currency units.

In accordance with our preregistered data analysis plan, we tested the differences between the control and the experimental conditions in round 2 using Wilcoxon rank sum tests with continuity correction. We repeated the analysis with less conservative Welch two sample* t* tests. Participants in the experimental condition had significantly riskier portfolios (*W* = 68,128, *P* < 0.001, *d* = 0.2; see Fig. 2A), and they used the decline in price to buy the risky asset. Therefore, our data clearly supports the first hypothesis.

We found weak support for Hypothesis 2a, which states that participants in the experimental condition will make more trades. The Wilcoxon test was not statistically significant (*W* = 75,591, *P* = 0.11) and had a weak effect size (*d* = 0.07), whereas the Welch test was significant (*t* = − 1.97, *df* = 561.33, *P* = 0.05). Further, participants in the experimental control condition showed a trend towards initiating larger transactions on average, partially supporting Hypothesis 2b. This difference was significant according to the Welch test (*t* = − 2.09, *df* = 797.51, *P* = 0.05) but not according to the Wilcoxon test (*W* = 75,512, *P* = 0.06), and the effect size was small (*d* = 0.1). The combined value expressed in experimental currency units of all shares bought and sold by a participant was significantly higher in the experimental condition than in the control condition (*W* = 72,374, *P* = 0.01, *d* = 0.13; *t* = − 2.55, *df* = 692.98, *P* = 0.01, see Fig. 2B), supporting Hypothesis 2c.

**Figure 3:**
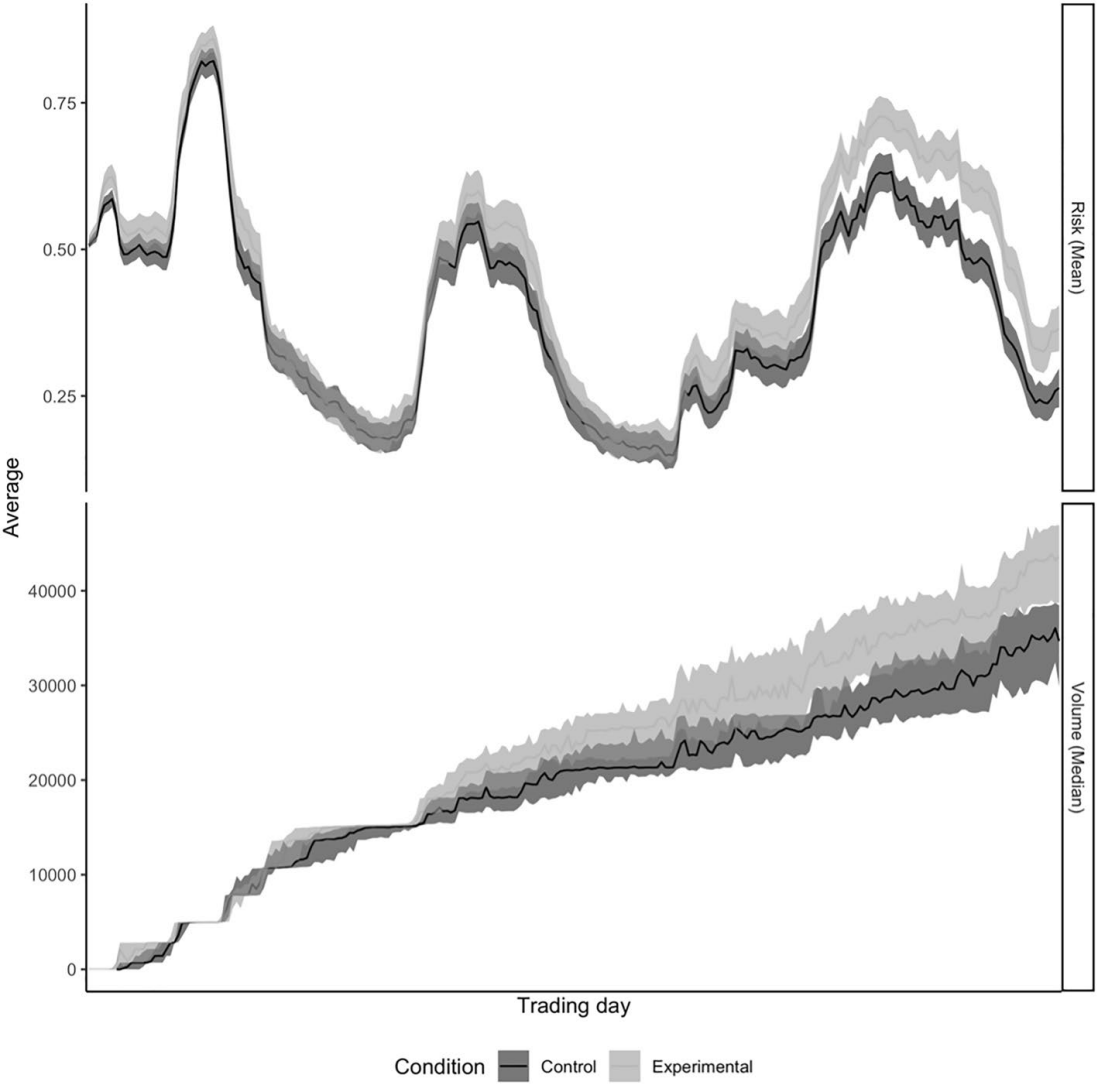
The development of mean portfolio risk and median Vol_Sum aggregated across participants. *Note* The solid line corresponds to the bootstrapped median. The shaded area around the solid line corresponds to the 95% bootstrapped confidence interval.

Descriptive statistics for all hypotheses are presented in Table 1. To verify that the effects that we report in this section are nonrandom patterns that became significant, we conducted a randomization test in which we shuffled the data 50,000 times by re-allocating the experimental condition to observed trading activity metrics. For each dependent variable tested in the hypotheses, we plotted the distribution of the *W* test statistics obtained from each reshuffling iteration in Fig. 3. We repeated this procedure with mean group differences Between conditions and compared this distribution to the difference observed between group medians. As Fig. 3 shows, the effects driving hypotheses 1 and 2c were due to differences between conditions starting from about experimental trading day 120 (one and a half minutes). Although an effect size of 0.13 is considered small^56^, a comparable real-world increase in US retail volume would correspond to about 600 million additional shares traded each day^57^.

**Table 1:** Descriptive statistics for the variables for testing differences between the control (C) and experimental (E) condition corresponding to each hypothesis.

Variable	Mean		Median		Min		Max	
	C	E	C	E	C	E	C	E
*H1: Risk*	0.39	0.44	0.37	0.42	0.01	0.04	0.99	0.99
*H2a: Trans*	21.53	25.00	18	20	0	0	159	453
*H2b: Vol*	13.92	14.67	15.67	16.13	1	1	20	20
*H2c: Vol_Sum*	41081	47860	34644	42974	0	0	321102	385437

Variables are explained in the main text. Variable *Vol* is related to the values of the quick-trade buttons (see Fig. 1A).

### Robustness checks

To ensure that the differences documented in round 2 were not attributable to a randomization failure such that pre-existing group differences might have been responsible for our observed effects, we tested the differences in risk-taking and trading activity in round 1, before the upward social comparison was induced. We used Wilcoxon tests on the differences between the control and experimental conditions, but found no differences in *Risk* (*W* = 78,335, *P* = 0.45), *Trans* (*W* = 79,793*, P* = 0.85*), Vol* (*W* = 82,020*, P* = 0.71) and *Vol_Sum* (*W* = 79,730, *P* = 0.74). Also, we found no relation between *Risk* and return on investment (ROI), as indicated by an insignificant Pearson’s correlations for round 1: *r* = − 0.01, *t* = − 0.41, *df* = 804, *P* = 0.68 and round 2: *r* = − 0.06, *t* = − 1.72, *df* = 804, *P* = 0.08.

Further, we found that participants’ earnings in neither round 1, nor round 2 differed between the conditions (Welch two-sample *t* test for *ROI*_1_: *t* = − 1.40, *df* = 776.89, *P* = 0.16, and for *ROI*_2_: *t* = − 0.99, *df* = 758.6, *P* = 0.32). This is to some extent to be expected given the absence of return predictability and low expected returns.

In addition to this, we tested for potential differences between participants in the control and in the experimental condition that could contribute to the differences in trading behaviour in round 2. According to Wilcoxon-sum rank tests, we found no differences in the self-reported propensity to take risk (*W* = 74,221, *P* = 0.07) and in three financial literacy questions (Question 1: *W* = 82,650, *P* = 0.28; Question 2: *W* = 78,634, *P* = 0.50; Question 3: *W* = 82,650, *P* = 0.28). The sample demographics were also similar with 39% of women and mean age of 40.01 years in the experimental condition and 36% of women and mean age of 41.10 years in the control condition indicating no statistical differences in age between the two conditions, *t* = 1.1235, *df* = 790.67, *P* = 0.26.

Finally, an alternative explanation for the observed differences in trading activity between the two conditions (Hypothesis 2c) is that this effect is driven entirely by differences in risk-taking (Hypothesis 1). This would happen if the additional trading activity in the experimental condition consisted exclusively of buying shares of the risky asset for the purpose of increasing portfolio risk. In such a case, an observed increase in trading volume would be mechanistically driven by an increase in risk appetite. To test this, we regressed *Vol_Sum* in round 2 on *Risk* in round 1 and a dummy variable for the experimental condition. Due to the distribution of *Vol_Sum*, we implemented a rank-based estimation method for linear models from the rfit R package^58^. The effect of condition on trading activity was substantially unchanged after accounting for concurrent differences in risk-taking (*β* = 0.058, *P* = 0.02). This implies that the higher trading activity observed in the experimental condition cannot be interpreted as an artefact or by-product of increased risk-taking.

Overall, these findings indicate that the social comparison induced in the experimental condition significantly increased risk-taking and trading activity.

### Manipulation check

After the trading task, we asked participants to estimate the mean performance of all participants to verify that participants in the experimental condition perceived typical earnings in the task as higher than those in the control condition. According to a Wilcoxon test (*W* = 51,600, *P* < 0.001), participants in the experimental condition perceived the average earnings of all participants, expressed in experimental currency, to be significantly higher than the participants in the control group (*Md*_*Control*_ = 500, *Md*_*Experimental*_ = 1000, *d* = 0.46). Therefore, we conclude that our manipulation was effective.

### Post-task satisfaction

In a preregistered analysis, we posed a question, ‘How do you feel about your performance in the trading task?*’,* adapted to our experimental design from^38^. In line with their results, we found that participants in the experimental condition were substantially less satisfied with their own performance than were participants in the control condition (*Md*_*Control*_ = 5, *Md*_*Experimental*_ = 4, *d* = 0.36, *W* = 1,022,889, *P* < 0.001).

## Discussion

Social trading platforms make financial asset trading easily accessible to anyone with internet access and some disposable capital. They also provide access to a large amount of information about the financial strategies and behaviour of traders. The COVID-19 pandemic has accelerated the growing popularity of online trading, with many people staying at home and channelling their time and effort into learning retail trading^3,59^. In 2020, the market size of online trading amounted to 8.28 billion USD, with growth to 12.16 billion USD forecast by 2028^60^ raising the question of how this growing trend impacts individuals. Relative performance information is omnipresent in the real-world markets, but laboratory studies systematically investigating its impact on retail investors are very scarce^38^.

To address the need to study human interaction with increasingly popular trading platforms, we investigated the influence of upward social comparison on risk-taking, trading activity and trading performance satisfaction with a dynamic trading task. Our results confirmed previous findings indicating that upward social comparison results in more risky trading behaviour^12,13,36,38,41^. Our findings add to previous research by experimentally demonstrating that upward social comparison also increases trading activity such as total traded volume. In contrast to the previous research demonstrating that upward social comparison increases trading activity^18,22,25^, we indicated three measures of trading activity which may allow us to identify whether the impact of social influence triggers affective or cognitive drivers of goal-directed behaviour. The trading behaviour we observed confirms the suggestion that even in financially literate investors, upward social comparison causes strategic trading behaviour to be moderated by affect; this corresponds to the psychological literature^44,46,61–64^.

Overall, participants were ultimately less satisfied with their performance, resonating with previous findings that more experienced investors derive more satisfaction from their own performance being ahead of others’^35,36,38,65,66^. This effect could be because the reward centre of the brain responds more strongly to a performance generated by skills and effort than one resulting from luck^66^. Given that previous experience with asset trading is linked to the expectation of performing well on social trading platforms^65^, financially literate participants of this study would perceive their performance as less rewarding when facing upward social comparison. Neuroscientific findings indicate that compared to no social comparison^67^ and downward social comparison^64^, upward social comparison is associated with increased brain activity in anterior insula, ventromedial prefrontal cortex (vmPFC), and dorsolateral prefrontal cortex (DLPFC), which are responsible for emotional processing (insula and vmPFC) and planning (DLPFC)^68^. The two conditions in our experiment did not differ in their final earnings, and, in contrast to tournament experiments, participants’ compensation was independent of that of other participants. We surmise that when participants trade extensively yet fail to perform as well as better traders, such participants may view their effort as wasted, lowering their trading satisfaction.

A limitation of the current study is that we deliberately did not investigate the effect of downward social comparisons as previous studies had^11,12,38^ and found that exposing participants to the worst investor’s performance led to less risky portfolio allocations. This decision was motivated by the fact that hardly any real-life social trading platforms show the worst performers. Moreover, success in finance and investment is much more salient than failure, and success stories are more viral than stories of loss^20,40^. Investment flows to the best-performing portfolio managers on social trading platforms^69^. Furthermore, early research on social comparison suggests an overall tendency of people to upward social comparison in performance situations^31,70^. Also, comparing oneself to richer people decreases people’s life satisfaction more than comparing themselves to poor people improves their life satisfaction^42^ indicating a smaller effect of downward social comparison in a financial setting^71^.

Further research could examine the impact of social influence on signal providers rather than on their followers. Signal providers should be considered as investment managers without direct access to the follower’s wealth^6^. Signal providers are more susceptible to the disposition effect—bias where an investor sells the well-performing assets, while keeping the badly-performing assets—than their followers, because they experience a fear of losing followers when admitting a poor decision^72^. Furthermore, the signal providers who receive more attention from their peers experience more excitement about trading, intensify their trading activity and increase risk-taking. Although the emotions and resultant increased trading activity wear off with time, the elevated risk-taking persists^73^, once again confirming the relation between social influence and trading behaviour. As a follow-up to this online experiment, future brain imaging and cognitive modelling studies could provide more in-depth insights to better understand the cognitive processes underlying the link between social comparison and trading.

Although previous studies showed no difference between using ECU and “real” currency^71^, another possible extension of this study would include upward social comparison including real rather than experimental currency. This would allow to capture the effect of social influence on investment behaviour in even more realistic setting. Related to this, future research could investigate the minimum difference between a person’s earnings and the top traders’ earnings required to induce change in trading behaviour. In addition to this, the current experimental design could be modified to offer copying trading strategies of the top performers, for the purpose of testing the impact of upward social comparison on copy-trading behaviour. Further extensions could involve modeling of trading behaviours and strategies and their change because of stimuli such as social influence. Potential models would account for differences in traders’ portfolio risk and trading activity and would allow for model fitting with experimental data for testing the validity of these models.

Nevertheless, findings from the current study are particularly relevant for the nascent yet rapidly expanding research devoted to social trading mechanisms and platforms. Recent European regulation MiFID II, Directive 2014/65^74^ aims to provide guidelines for disseminating only ‘fair, clear and not misleading’ materials to prospective investors, but it provides no explicit guidelines for presenting peer information either as part of marketing messages or integrated into platforms’ user interfaces. This study shows that presenting the returns of top performers can increase other traders’ risk-taking and effort. Excessive trading, which usually occurs on online trading platforms, leads to mental health problems in some users^75^. Presenting only the best-performing portfolios on social media platforms promotes herding in trading strategy selection, in which past winners are bought more frequently and past losers are more frequently sold^76^. However, followers often obtain worse performance than the leaders who initially implement strategies^14^. In contrast, open information exchange among users of social trading platforms could promote self-consciousness and reduce biases such as the disposition effect^8^.

In sum, this study contributes to the current literature on social influence and trading behaviour by demonstrating that social comparison impacts trading activity on an online trading platform. In a naturalistic experiment with financially literate participants, it investigates three, linked with each other, aspects at a time: risk-taking, three facets of trading activity and traders’ satisfaction. It demonstrates that investigating various aspects of user activity on online trading platforms can help better understand the psychological triggers of user behaviour and potential biases. Further experimental research on how people respond to online and offline social influences in the domain of finance, and how new technologies facilitate these effects, should inform future policy efforts to maximize consumer welfare. It is necessary for behavioural research to facilitate safe market participation of the retail investors—regular members of our society investing their savings. We believe that this study provides a steppingstone towards a better understanding of the interplay between social and technological factors influencing retail investing.

## Method

The study was preregistered on the Open Science Framework (https://osf.io/we48d/) and approved by the ETH Ethics Committee (2020-N-120). Anonymized data, analysis code and experimental materials are available on the Open Science Framework. All methods were performed in accordance with the relevant guidelines and regulation. This study uses a difference-in-difference, mixed experimental design with the upward social comparison as an independent between-subject variable and trading behaviour (risk and trading activity) measured as both within and between dependent variables.

### Participants

In the period 17.11.2020–07.12.2020, we recruited 1622 CloudResearch Mechanical Turk workers, aiming to collect data from 800 participants, based on an a priori power analysis. 1262 individuals entered the experiment and accepted the informed consent form, and after the prescreening, 807 people (300 female, *M*_age_ = 40.62, Age range: 18–83) completed the full study. This resulted in a 64% pre-screening rate for consenting participants. Each prospective participant received 0.30 USD base payment regardless of whether they passed the initial screening, and everyone who completed the experiment received a variable performance-dependent bonus ranging from 1.38 to 1.66 USD (*M*_Bonus_ = 1.51). The participants had to be verified by Amazon as individual investors directly holding investments in the US stock market. Their investor status had been previously checked by Cloud Research^77^, they were identified as ‘high-quality’ based on their earlier survey participation, and they had completed more than 100 human intelligence tasks with an acceptance rate of at least 95% on Mechanical Turk. Further participant selection criteria included completing the experiment using Chrome browser to ensure the best possible compatibility with our experiment platform, being a resident of the United States and being physically present in the United States at the time of data collection.

### Materials and procedure

We sent out invitations to a pool of workers fitting our recruitment criteria in 13 batches to reach potential participants at different times. After accepting the invitation, a worker was re-directed to a link to Qualtrics, an online survey platform, which randomly assigned them to one of the two conditions: the control condition and the upward social comparison condition. Participants in both conditions underwent four steps: (1) a pre-screening questionnaire for investing on the stock market and financial literacy, (2) a self-reported risk attitude measure, (3) the Zurich Trading Simulator trading task (c.f., ZTS,^78^) and (4) a short questionnaire about participants’ self-evaluation of their performance relative to others’ and a few demographic questions.

The prescreening questionnaire consisted of 11 questions (see the Online Appendix): three questions were filler questions, and eight questions inspired by^79^ were designed to screen out people with insufficient knowledge of and experience in finance. Three additional questions^80^ were implemented after the screening was complete to ascertain that passing the screener is indeed associated with higher financial knowledge. The screening procedure helped us avoid those overclaiming by falsely stating the possession of certain skills or attributes, which is a serious concern with the validity of data collected on online labour markets^81^. An example question prompted prospective participants to indicate whether they currently hold or recently have held a direct investment in any of the companies listed as answer options; however, all the widely-known consumer brands listed were privately held at the time of data collection. Thus, selecting any option other than ‘none’ would indicate overclaiming. A pilot analysis of an early batch indicated that participants who passed the screening exhibit significantly higher objective financial literacy than those who were screened out (*t*(141), *P* < 0.001). We observed the same effect in the final experimental dataset (*t*(136.82), *P* < 0.001).

Once a participant had passed the prescreening questionnaire, they were asked to provide informed consent by signing a form. If they consented to participate in the study, their self-reported attitude towards risk was measured with the SOEP German Socio-Economic Panel general risk item^82^.

Next, participants were redirected from Qualtrics to the trading task (Fig. 1B displays the user interface of the task), which consisted of one practice round to explain the functionality of the software and the task and two experimental rounds. The practice round presented an artificial price pattern accompanied by an interactive introduction to the trading task, and the two experimental rounds included historical market index closing prices from the Swiss Market Index (SMI). We selected price paths such that they were broadly representative of the SMI’s performance and volatility profile over the past 20 years, but did not contain extreme events which might bias or inform participants about the future development of the price paths or induce wealth effects^83^. We standardized the historical prices to start uniformly at 141.7 ECU to ease comprehension and avoid any participant correctly identifying the source of the price path. The choice of the starting price was motivated by the conversion from the experimental currency to the real currency in USD, and by the values of quick-trade buttons (1, 5 and 20 shares) in the ZTS trading task.

Participants in the social comparison condition received information about the performance of three other participants in the study (see Fig. 1A). These participants were the best performers from the first batch of data collection, which only included participants from the control condition. In each experimental round in both conditions, participants were endowed with 10,000 units of experimental currency equally split between a risky stock and cash. After the second round of the trading task, the amount of money participants made in both rounds separately was summed, and participants were compensated for their cumulative performance. The accumulated earnings were converted from the experimental currency to USD at the exchange rate of $1 for every 14,000 in experimental currency earned and paid out as a bonus. Before participants entered the trading task, they were randomly assignment to either control or experimental condition using Qualtrics randomization tool.

After completing the trading task, participants were directed back to Qualtrics to complete the post-trading questionnaire including two questions: one measuring participants’ perception of the average performance of all participants in a manipulation check and one measuring participants’ satisfaction with their own performance. The post-trading questionnaire also included demographic questions and questions about experience in and knowledge of investing in finance. At the end of the study, each participant was provided with their unique MTurk exit code, which entitled them to receive payment, and was then thanked for their participation.

### Supplementary Information

Supplementary Information.

## Data Availability

Data is freely available on Open Science Framework (https://osf.io/we48d/).
