# Supplementary material for: The influence of upward social comparison on retail trading behaviour
Source: Sci Rep. 2023 Dec 19;13:22713. doi: 10.1038/s41598-023-49648-3 (PMC10733348; doi:10.1038/s41598-023-49648-3)
Supplement: Supplementary file 1 — Supplementary Information. [file 41598_2023_49648_MOESM1_ESM.docx]

**Appendix**

**A: *Screenshots with Examples of Real-life Social Trading Platforms*** *presenting peer information on two famous ZuluTrade and eToro. Any personal and identification information is hidden.*

**
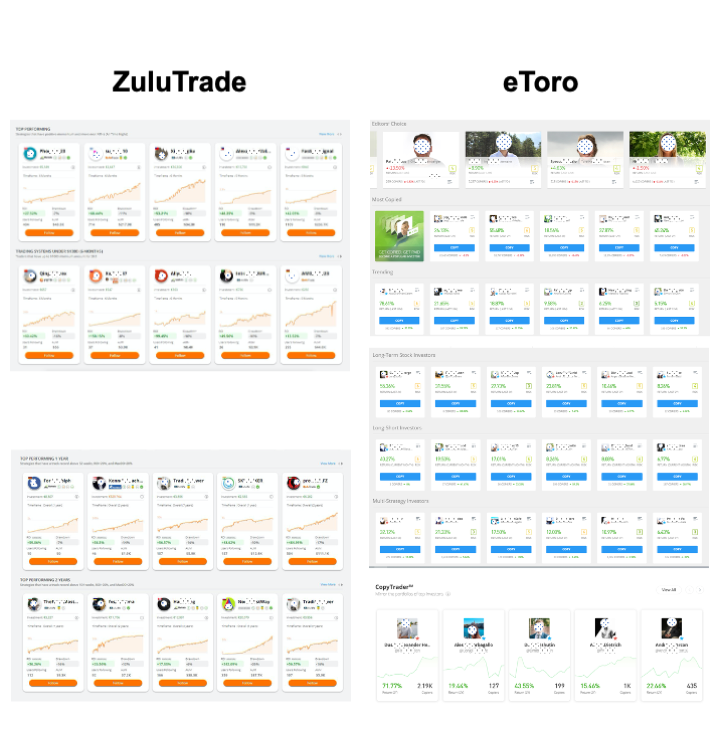
**

**B: *Key Definitions*** *frequently used in the manuscript*

**Affect** is defined as “any experience of feeling or emotion, ranging from suffering to elation, from the simplest to the most complex sensations of feeling, and from the most normal to the most pathological emotional reactions. […] Both mood and emotion are considered affective states. Along with cognition and conation, affect is one of the three traditionally identified components of the mind.” (American Psychological Association, APA Dictionary of Psychology, <https://dictionary.apa.org/affect>, last accessed, 17.08.2022)

A **cognitive process** is defined as “any of the mental functions assumed to be involved in the acquisition, storage, interpretation, manipulation, transformation, and use of knowledge. These processes encompass such activities as attention, perception, learning, and problem solving.” (American Psychological Association, APA Dictionary of Psychology, <https://dictionary.apa.org/cognitive-process>, last accessed 19.07.2022).

**C: *Pre-Screening Questionnaire*** *with correct answers included in [the square brackets]. Filler questions marked with (F), the pre-screening questions are marked with (S), while the financial literacy are marked with (L).*

*Filler questions are not used for the pre-screening and any answer in the filler questions is correct.* *The point of the pre-screening questions was to screen out participants who are not actively investing in the stock market. The point of the financial literacy questions*

*The following questions were presented in a randomized order. In order to pass the pre-screening questionnaire, a participant had to correctly respond to all pre-screening and financial literacy questions.*

1. How many hours a week do you exercise? (F) [all answers are correct]
   1. I generally don’t exercise
   2. 20 minutes to 1 hour
   3. 1 hour – 2 hours
   4. 2 hours – 3 hours
   5. More than 3 hours
   6. I’d prefer not to say
2. Which of the following best describes your current status? (F) [all answers are correct]
   1. Student
   2. Employee
   3. Home maker
   4. Business owner
   5. Unemployed
   6. Retired
   7. Other
   8. I’d prefer not to say
3. Are you registered to vote? (F) [a participant should answer ‘a’ to pass the screening]
   1. Yes
   2. No
4. Do you currently hold a US passport? (S) [a participant should answer ‘a’ to pass the screening]
   1. Yes
   2. No
5. Do you personally invest in the stock market? (S) [a participant should answer ‘a’ to pass the screening]
   1. Yes
   2. No
6. Do you currently hold an investment in the stock market? (S) [a participants should answer ‘a’ to pass the screening]
   1. Yes
   2. No
7. Please, name one asset that is currently in your investment portfolio! (S)

{open question}

1. Please, tell us how your {asset name entered in question 4} investment performed over the past month in percent terms (rounded to the closest 10%) (S) [‘e, f, g, h, i, j, k, l, m’ are all correct answers. Any answer between -40% and +40% is acceptable. The point is to determine if people have an approximate idea of what is realistic performance.]
   1. < -70%
   2. -70%
   3. -60%
   4. -50%
   5. -40%
   6. -30%
   7. -20%
   8. -10%
   9. 0%
   10. 10%
   11. 20%
   12. 30%
   13. 40%
   14. 50%
   15. 60%
   16. 70%
   17. <70%
2. Which of the following companies do you own stock in directly, or have done so in the past 3 months? (S) [‘j’ is the correct answer]
   1. Panera Bread
   2. Mars
   3. State Farm
   4. Liberty Mutual
   5. Pilot Travel Centers
   6. Mass Mutual
   7. Penske
   8. Publix
   9. Albertsons
   10. None of the above
3. Please select which brokerage service provider you use to invest in stocks! (multiple answers are possible) (S) [‘a’ or ‘g’ are the correct answers]
   1. Interactive Brokers
   2. TradeFox
   3. E-Stox
   4. Forex.com
   5. Wall Street Associates
   6. Comdirect
   7. Other
4. Please name the brokerage service that you are using to invest in stocks! (S) [any answer is correct]

{open question}

1. What has been the average annual return on a very broad US stock index market investment over the last two decades, in percentages? (S) [any value in the range from 5 to 10 is correct]

{open question}

1. Which of the following statements describes the main function of the stock market? (S) [‘c’ is the correct answer]
   1. The stock market helps predict stock earnings
   2. The stock markets result in an increase in the price of stocks
   3. The stock market brings people who want to buy stocks together with people who want to sell stocks
   4. None of the above
2. Which of the following statements is correct? If somebody buys a bond of firm B: (S) [b is the correct answer]
   1. He owns a part of firm B
   2. He has lent money to firm B
   3. He is liable for firm B’s debts
   4. None of the above
3. Please complete the Captcha below to show that you are not a robot! (S) [the captcha is automatically generated by Qualtrics]

**D: *Informed Consent form***

I have been informed about the aims and procedures of the study, the advantages and disadvantages, as well as potential risks of participating.
I have read and understood the information sheet for volunteers (included above).
I was given sufficient time to make a decision about participating in the study.
I agree that the responsible investigators and/or the members of the Ethics Committee of ETH Zurich have access to the original data under strictly observed rules of confidentiality. I participate in this study on a voluntary basis and can withdraw from the study at any time. I recognize that participants who exit the study before its conclusion forfeit their right to compensation.

**By clicking ‘Proceed with the study’, I agree to all of the statements above, and agree to participate in the study:**

1. Proceed with the study
2. Exit study

**D: *Self-reported risk attitudes***

How do you see yourself? Are you generally a person who is fully prepared to take risks or do you try to avoid taking risks?

- 1. Not at all willing

  4. Moderately willing

  7. Very willing

**E:** ***General instructions to the experiment***

**A study on how individuals behave on a simulated trading platform**

The goal of the present study is to investigate how individual investors manage their investments.
The study consists of a trading task, followed by a short questionnaire.

You will receive 10 000 Experimental Currency at the beginning of each round of a realistic trading simulation.
**Your performance in each of the two rounds will be added up, and paid out in real money.**

For every 14 000 in Experimental Currency you earn, you receive $1 in real money.
The more you make in the task, the more real money you earn.
A person earning 0% in both rounds can expect to earn a bonus of $1.43.
You will also receive a guaranteed base payment of $0.30 for having participated, regardless of your performance. 

The median expected completion time of the study is **14 minutes**.

If you encounter problems submitting this HIT, please email [dkaszas@ethz.ch](mailto:dkaszas@ethz.ch).

Further terms and information:

It will not be possible to connect your identity to the information you provide us in your survey answers.
Only the responsible investigators and/or the members of the Ethics Commission will have access to this anonymous data under strictly observed rules of confidentiality.
Please be aware that participating in this study does not present any known risks to participants, physical, psychological or otherwise.
You are not obliged to complete the study, and can quit anytime without having to justify it to the requester.
However, due to the design of our study, only participants who have completed the whole study can claim their compensation.

Possible damage to your health, which is directly related to the study and demonstrably the fault of ETH Zurich, is covered by the general liability insurance of ETH Zurich (Insurance Policy No. 30/4.078.362 of the Basler Versicherung AG).
Beyond the aforementioned conditions, health insurance and accident insurance is the responsibility of the participant.
The study is financed by the Dr. Donald C. Cooper-Fonds.

**F:** ***Instructions to the ZTS trading task***

**Instructions**

The upcoming trading task will be conducted using a browser-based trading interface (see the image below for an illustration).

The task consists of three rounds: one practice, and two main rounds.
Your combined performance in the two main rounds determines your compensation.

At the beginning of each round you will be endowed with an initial portfolio consisting of shares of a risky asset (Shares) and a safe asset (Cash), worth a combined 10 000 currency units.
50% of this endowment will be in the form of the risky asset (Shares), and 50% will consist of the safe asset (Cash).
In each round, you can buy or sell Shares by clicking ‘Buy’ or ‘Sell’ buttons of the corresponding size.
Shares are characterized by higher volatility than Cash. 

The prices are historical daily closing prices of a real-world market index.
Prices are predetermined, and your actions do not have an influence on market prices.

Any trade that you make will be executed instantly at the current market price.
You can buy or sell as many shares as you want, under the condition that you have sufficient funds for the transaction.
No short-selling is possible.

The y-axis of the price chart adjusts automatically to the current market price.

These changes only reflect the current price, and are not informative for future price changes.


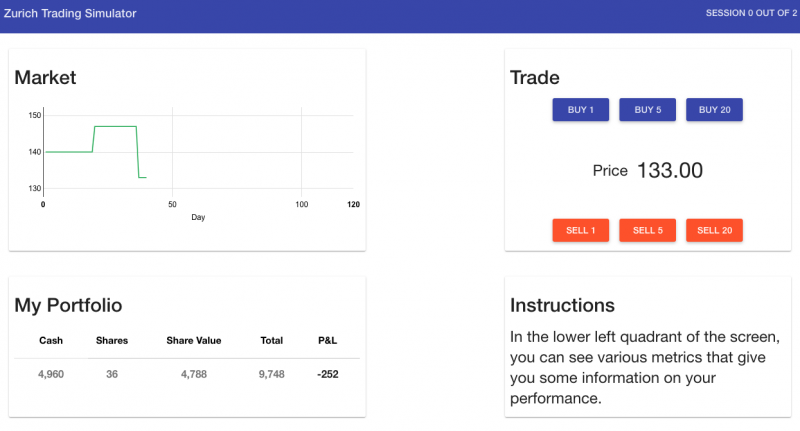


**Other people also participate in this study, but you do not interact with them.**

**Depending on which study condition you are in, you might see information on how some of them have performed.**

**G: *Explanation of the ZTS task***

**This is the practice round.**Its goal is to familiarize you with the trading software.
Your performance in this round does not have an influence on your compensation.

Please pay attention to the instructions appearing in the box in the lower right corner!

***The following information was displayed in the ‘Instructions’ box***

1. In order to achieve the best performance possible in the upcoming experimental round, pay attention to the instructions.
2. In order to make trades, you can use the six buttons above to buy or sell different amounts of stock.
3. The current price of the asset will be presented between the two rows of buy and sell buttons.
4. In the lower left quadrant of the screen, you can see various metrics that give you some information on your performance.
5. The first metric is Cash. It tells you how much liquidity you currently have that you can use to buy shares.
6. The second metric is Shares. It tells you how many shares are currently in your portfolio.
7. The third metric is Share Value. It tells you how much the shares in your portfolio are currently worth.
8. The fourth metric is Total. It is the combined value of your Cash and Shares.
9. The fifth metric is Profits and Losses. It tells you how much money you have made or lost, compared to the amount of money you started the round with.
10. Try buying and then selling 20 shares to demonstrate you have understood the instructions.
11. On the screen following the first round, you can find an overview of how you performed.

**This is the first round.
Your performance from now on will determine your compensation.
There are other people participating in this study.
After this round, you will receive performance feedback.
You will either see only your performance, or your performance and the performance of others.**

**H: *Post-trading task questionnaire***

1. Please, give us an estimate for how much money you think other participants earned in the **first main round** on average! (in P&L term, relative to the starting portfolio value of 10 000) The mean participant earned…

{open question}

Experimental Currency Units

1. How do you feel about your performance in the trading task? (Overall, across both main rounds)
   1. 1 - Very negatively
   2. 2
   3. 3
   4. 4 – Neutral
   5. 5
   6. 6
   7. 7 – Very positively
2. Gender
   1. Male
   2. Female
   3. Other
3. Age

{Open question}

1. Highest level of education completed:
   1. Less than high school diploma
   2. High school diploma or GED
   3. Some college, but no degree
   4. Associates degree
   5. Bachelor’s degree
   6. Master’s degree
   7. Professional degree
   8. Doctorate
   9. Other: {Open question}
2. How would you judge your own level of experience in investing?
   1. 1 - No experience
   2. 2
   3. 3
   4. 4 – Moderate experience
   5. 5
   6. 6
   7. 7 – Very experienced
3. How would you judge your knowledge and understanding of finance and investments?
   1. 1 – No knowledge and understanding
   2. 2
   3. 3
   4. 4 – Moderate
   5. 5
   6. 6
   7. 7 – Very high knowledge and understanding
4. Please, share any observations you have regarding the study! (optional)

{Open question}

**I: *Final information provided to participants***

**Checkout**

You have finished the study. Thank you for taking the time! In order to receive your payment you must copy and paste the following code back to Amazon Mechanical Turk:

**{9-digit MTurk code}**

Your payment will be processed within the next 48 hours. If you encounter problems submitting this HIT, please contact dkaszas@ethz.ch and report the problem there.

Thank you!

**I: *Price patterns displayed in the ZTS trading task***

| ***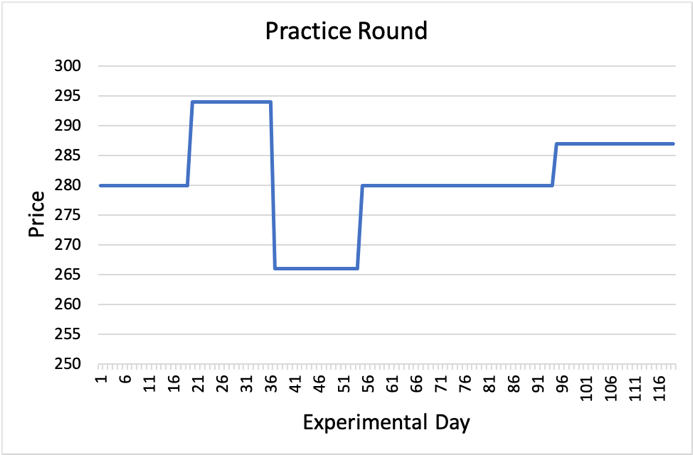*** | |
| --- | --- |
| ***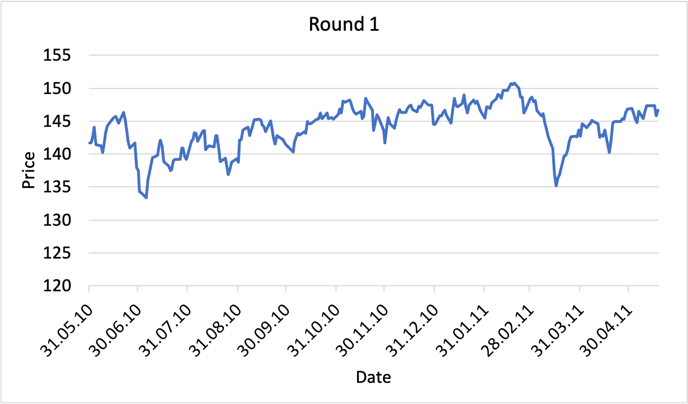*** | ***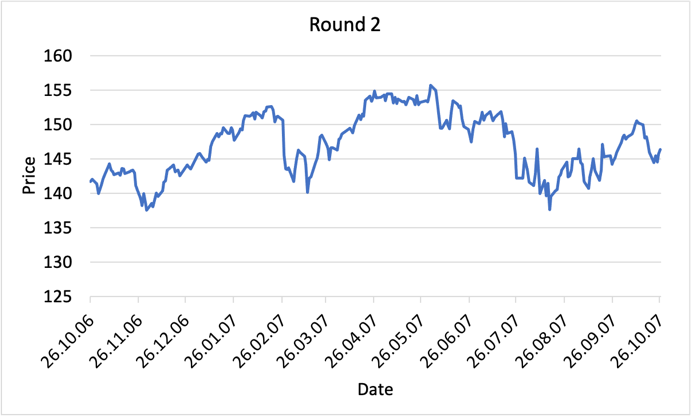*** |

*Figure A1.* Price charts used in the practice round, the first and the second experimental round. The price data for the practice round were artificially generated such that they do not prime participants with any price patterns. Data in experimental rounds 1 and 2 are historical closing prices from the Swiss Market Index (SMI) 31.05.2010 – 18.05.2011 and 21.10.2006 – 26.10.2007.
